# Addressing Stress and Rhythm Dysregulation among Health Workers in the Post-Pandemic Context: An Italian Study and a Proposal for a Preventive Psychoeducational Intervention

**DOI:** 10.2174/0117450179449051260114063307

**Published:** 2026-02-27

**Authors:** Elisa Cantone, Antonio Urban, Cesar Aviles Gonzales, Alessandra Perra, Stefano Lorrai, Michela Atzeni, Stocchino Serena, Rober Romero Ramírez, Adalberto Rangel Restrepo, Mauro Giovanni Carta, Giulia Cossu

**Affiliations:** 1 Capacities Building for Global Health, University of Cagliari, 09124 Cagliari, Italy; 2 Department of Medical Sciences and Public Health, University of Cagliari, 09124 Cagliari, Italy; 3 Alumni Group of Nursing, Universidad Popular del Cesar, Valledupar 200002, Colombia; 4 Department of Medicine and Surgery, University of Enna “Kore”, Piazza dell’Università, 94100 Enna, Italy

**Keywords:** Healthcare workers, DYMERS, COVID-19, Rhythm dysregulation, Prevention, Depressive symptoms

## Abstract

**Introduction:**

During the COVID-19 pandemic, healthcare workers (HCWs) faced intense and prolonged stress. This exposure increased their risk of burnout and mood disorders. The *Dysregulation of Mood, Energy, and Social Rhythms (DYMERS) hypothesis* suggests that instability in biological and social rhythms may precede the onset of mood dysregulation. This study examined rhythm disturbances among HCWs and explored the potential role of a rhythm-focused psychoeducational intervention.

**Methods:**

Ninety-seven HCWs from the University Hospital of Cagliari, Italy, participated in a cross-sectional study. Depressive symptoms were assessed using the Patient Health Questionnaire-9 (PHQ-9), and rhythm regulation was evaluated through the Biological Rhythms Interview of Assessment in Neuropsychiatry (BRIAN). Data were compared with published findings from mood disorders, psychotic disorders, and community samples.

**Results:**

A current depressive episode with PHQ-9 score was identified in 35.1% of HCWs participants, with significantly higher prevalence (*p*= 0.01) among women (42.7%) than men (14.7%). Mean BRIAN scores were 46.1 ± 11.0, significantly higher than those observed in psychotic disorder (*p* = 0.04) and community samples (*p*< 0.0001). Rhythm dysregulation scores approached values reported in bipolar disorder samples. Elevated BRIAN scores were also observed among non-depressed participants.

**Discussion:**

The elevated rhythm dysregulation scores observed in healthcare workers support the hypothesis that chronic occupational stress may disrupt biological and social rhythms, potentially increasing vulnerability to mood disorders.

**Conclusion:**

HCWs displayed pronounced rhythm dysregulation and high rates of depressive symptoms, highlighting occupational stress as a potential major risk factor and further supporting the DYMERS hypothesis. From both clinical and research perspectives, it could be essential to promote psychoeducational programs that target rhythm regulation, such as adaptations of Interpersonal and Social Rhythm Therapy, which may offer effective preventive strategies to enhance resilience and mental well-being in this population. However, the cross-sectional design, convenience sampling, limited socio-demographic data, and reliance on self-reported measures constrain generalizability and causal inference, underscoring the need for larger longitudinal studies to validate and extend these findings.

## 1 INTRODUCTION

The *Social Zeitgeber Hypothesis* (from the German *zeitgeber*, “time giver”) proposes that disruptions in social routines can destabilize biological rhythms [1]. These routines include sleep–wake cycles, mealtimes, and interpersonal interactions. In vulnerable individuals, such disturbances may trigger mood episodes [2, 3].

Evidence shows that life events disrupting daily routines and sleep instability often precede depressive or (hypo)manic episodes [4, 5]. Similarly, sleep instability and circadian disruption are commonly observed in individuals with Bipolar Disorder (BD) even during euthymic phases [6-8]. Importantly, therapeutic approaches designed to stabilize rhythm, such as Interpersonal and Social Rhythm Therapy (IPSRT), have demonstrated efficacy in improving remission and psychosocial functioning [9, 10]. Collectively, these findings emphasize rhythm regulation as a critical mechanism in both the onset and management of BD. Building on this framework, the concept of DYMERS (“Dysregulation of Mood, Energy, and Social Rhythms Syndrome”) was recently introduced [11]. A preclinical condition is characterized by behavioral rhythm dysregulation that may increase vulnerability to mood disorders depending on individual stress exposure. DYMERS has been linked as a transdiagnostic vulnerability factor. It could affect clinical populations with different disorders such as mood disorders, panic disorder, attention deficit/ hyperactivity disorder (ADHD), and post-traumatic stress disorder (PTSD) [12-14]. However, it could also affect high-risk groups, including healthcare workers (HCWs) and individuals with chronic illnesses, who may experience burnout and other stress-related outcomes [15-17]. This underscores the broader clinical relevance of rhythm dysregulation in vulnerable populations.

HCWs are particularly vulnerable to rhythm dysregulation due to night shifts, unpredictable schedules, understaffing, and organizational strain. Evidence shows that misalignment between internal circadian rhythms and work schedules increases the risk of burnout, depression, and poor sleep, independent of shift type [18-22]. The COVID-19 pandemic further exacerbated occupational stress [23], making HCWs an ideal population to investigate the presence and impact of rhythm disruption in a non-clinical, high-risk context.

### 1.1 Study Aim

Against this background, the present study aimed to investigate mood and rhythm dysregulation in HCWs, a population at high risk for mental health difficulties due to post-pandemic stress, workload, and organizational instability

The specific objectives were to:

1. Assess the prevalence and severity of depressive symptoms among HCWs using a standardized self-report instrument.

2. Evaluate the degree of biological rhythm dysregulation in the same sample through a validated international measure.

3. Examine the relationship between depressive symptoms and rhythm dysregulation, exploring whether greater rhythm instability is associated with current depressive episodes.

4. Compare rhythm dysregulation levels in HCWs with those previously reported in clinical samples with bipolar and psychotic disorders, and in a community sample from the same geographic area.

5. Discuss the implications of these findings for the development of rhythm-focused psychoeducational interventions aimed at prevention and early support in this high-risk occupational group.

In this non-clinical but high-risk population, we hypothesize that HCWs will exhibit biological and social rhythm dysregulation at levels comparable to clinical populations with mood disorders. The co-occurrence of depressive symptoms among those with rhythm dys-regulation may indicate increased vulnerability for future mood episodes, consistent with the Social Zeitgeber and DYMERS frameworks. Given the cross-sectional design, no causal conclusions can be drawn, but identifying both rhythm dysregulation and depressive symptoms provides important heuristic insight for future longitudinal and preventive studies.

## 2 METHODS

### 2.1 Design

This study employed a cross-sectional design to investigate rhythm dysregulation and depressive symptoms among HCWs. Participants were recruited from four outpatient facilities affiliated with the University Hospital of Cagliari, Italy, providing a representative sample of health professionals working in diverse clinical settings. Voluntary convenience sampling will be conducted based on the availability of eligible participants at each recruiting center, following verification of inclusion criteria and acquisition of informed consent. The study was conducted and reported in accordance with the STROBE statement guidelines for observational studies [24].

### 2.2 Study Sample

Participants eligible for inclusion in the study were adults aged 18 years and older, of any gender, who provided signed informed consent. Eligible healthcare workers were those professionally engaged in care services within specified categories, including physicians, nurses, psychologists, healthcare assistants, social workers, and rehabilitation staff. Individuals who did not meet these inclusion criteria or who had severe medical or psychiatric conditions that would prevent them from completing the study questionnaires were excluded. Data collection occurred during a single week in May 2025 to capture a consistent snapshot of participants’ status. The sample included 97 HCWs, comprising both fully qualified staff and trainees who, according to Italian regulations, are authorized to perform clinical duties during their final postgraduate years. This approach ensured that all participants had active responsibilities in healthcare delivery, making the findings relevant to real-world clinical practice. Given the voluntary nature of participation, a potential self-selection bias cannot be excluded, as individuals experiencing higher levels of stress or workload may have been less likely to participate.

### 2.3 Study Instruments

Following participants’ signing of informed consent, which was approved by the local ethics committee, the following validated instruments were administered:

#### 2.3.1 Demographic and Work-related Questionnaire

This tool collected essential background information, such as age, gender, employment ward, and professional role, to characterize the sample and explore potential associations with study outcomes. Some socio-demographic data, such as years of service and type of work shifts, could represent potential sources of bias, since participant recruitment was conducted within a single healthcare organization. For this reason, collecting such data might have risked compromising participant anonymity and violating privacy principles.

#### 2.3.2 Patient Health Questionnaire – 9 item (PHQ-9)

The Italian version of this widely used self-report scale [25, 26] was utilized to screen for depressive episodes. The PHQ-9 aligns with DSM-5 diagnostic criteria by assessing nine core depressive symptoms. Scores range from 0 to 27, with a cut-off of 10 or above indicating a high probability of clinically relevant depressive symptoms, typically reflecting mild to moderate depression severity. This tool's psychometric properties and clinical utility are well established internationally.

#### 2.3.3 Biological Rhythms Interview of Assessment in Neuropsychiatry (BRIAN)

To evaluate disruptions in biological and social rhythms, we used the Italian validated version of BRIAN [27, 28]. This semi-structured interview assesses five key rhythm domains: sleep patterns, physical activity, social interactions, meal timing, and chronotype (a measure of an individual's preferred timing of daily activities). The questionnaire consists of 21 items rated on a four-point scale (1 = “not at all” to 4 = “often”), with higher scores indicating greater rhythm dysregulation. BRIAN has demonstrated strong reliability and has been translated and applied across multiple cultural contexts [29, 30].

### 2.4 Statistical Analysis

All data analyses were performed with IBM SPSS Statistics software (version 28.0.1, IBM Corp., Armonk, NY, USA. Descriptive statistics were calculated to summarize participant characteristics and study variables. Categorical variables were analyzed using Chi-square tests with Yates’ correction when necessary, and non-parametric methods were applied when distributional assumptions were not met. Normality of continuous variables (BRIAN scores) was verified through the Shapiro–Wilk test. Given approximate normality, parametric analyses were performed using one-way ANOVA with Bonferroni post-hoc tests, reporting Cohen’s d as the effect size for pairwise comparisons. To account for demographic differences, BRIAN scores were adjusted for age and sex prior to comparison with published reference samples. Differences between the present sample and previously published samples were then statistically tested using independent-sample t-tests.

## 3 RESULTS

A total of 97 HCWs completed the study assessments and were included in the sample. Two invited workers (one nurse and one medical doctor) refused participation (2.1% of the overall sample), citing pressing work commitments that prevented them from completing the study instruments. The sample represents a group of HCWs experiencing significant psychological distress (Table **1**). Specifically, thirty-four participants (35.05%) met criteria for a current depressive episode. No significant differences emerged between participants above and below 50 years of age. Women showed a higher prevalence of depressive symptoms compared to men, reaching statistical significance (χ^2^ = 5.76, *p* = 0.016), while non-physicians exhibited a non-significant trend toward higher depression rates than physicians (48.9% *vs*. 22.9%, *p* = 0.059). The mean BRIAN score for the sample was 46.14 ± 10.96, indicating moderate biological rhythm dysregulation. Women had higher mean scores than men (47.64 ± 12.82 *vs*. 42.62 ± 9.68), but the difference did not reach statistical significance (p *p*= 0.059). Participants with current depressive episodes reported markedly higher BRIAN scores compared to participants without current depressive episodes (55.12 ± 7.94 *vs*. 41.29 ± 12.58; *p* < 0.001; *d* = 1.3).

When compared with previously published data from the same geographic area (Table **2**), the mean BRIAN score observed among HCWs did not significantly differ from that of individuals with bipolar disorder reported in two independent studies (*p* = 0.536; *p* = 0.109). Conversely, it was significantly higher than that observed in a sample of individuals with stabilized psychosis (43.70 ± 10.36 *vs*. 40.40 ± 7.02; *p* = 0.043; *d* = 0.37) and markedly higher than in a community sample (44.77 ± 11.59 *vs*. 22.22 ± 11.19; *p* < 0.001; *d* = 1.97).

## 4 DISCUSSION

The findings of this study reveal a concerning degree of biological rhythm dysregulation among HCWs, a population that remains understudied within the context of rhythm-related psychopathology [34, 35]. Notably, the mean BRIAN scores observed in this non-clinical yet highly stressed group closely resemble those reported in individuals diagnosed with BD in previous research. Although the mean rhythm dysregulation observed among HCWs approached the levels reported in clinical populations, this should not be interpreted as diagnostic equivalence. Instead, these findings suggest a subclinical rhythm vulnerability consistent with the DYMERS framework, rather than a fully prodromal condition [36, 37]. The high prevalence of current depressive episodes (35.05%), particularly among women, aligns with global trends in post-pandemic mental health and underscores the central role of rhythm instability in mood deterioration [38-40]. These results support the conceptualization of rhythm disruptions not merely as secondary manifestations of mood disorders, but as core transdiagnostic mechanisms that may precede the onset of formal psychiatric diagnoses [13].

Furthermore, BRIAN scores in our sample exceeded those reported for individuals with psychosis in remission [31] and were markedly higher than those of a general community sample [41, 42]. This suggests that occupational stress and chronic disruption of daily routines may significantly impair rhythm regulation. Notably, the presence of substantial rhythm dysregulation among participants without current depressive episodes points to a latent, subthreshold dysfunction that may represent an early expression of rhythm instability within the DYMERS continuum.

Consistent with this interpretation, our results can be conceptually situated within a stress–rhythm dysregulation–DYMERS–mood disorder pathway model, whereby chronic stress exposure contributes to rhythm desynchronization, which in turn increases vulnerability to mood dysregulation. Future longitudinal work could visually summarize this conceptual pathway to better illustrate the progression from occupational stress to potential clinical outcomes.

### 4.1 Implications for Research

Our study provides heuristic evidence for rhythm dysregulation and its co-occurrence with depressive symptoms in HCWs, but the cross-sectional design precludes causal inference. Longitudinal and experimental studies are needed to clarify whether rhythm dysregulation represents a true risk factor or an early manifestation of emerging mood pathology. Future research should also explore the scalability, cultural adaptability, and efficacy of rhythm-based interventions, and examine potential moderators such as individual vulnerability, work schedules, and organizational context.

### 4.2 Implications for Clinical Practice

The clinical implications of our findings are considerable. Research on preventive strategies targeting rhythm dysregulation is still limited, with only a few small pilot studies available [41, 42]. Our study supports the concept of DYMERS as a prodromal syndrome characterized by rhythm dysregulation, which may represent a relevant clinical target in high-risk populations such as healthcare workers (HCWs). Given the robust evidence for Interpersonal and Social Rhythm Therapy (IPSRT) in stabilizing daily rhythms and reducing relapse in BD [43-45], adapting rhythm-focused psychoeducational interventions to HCWs appears promising. This alignment reinforces the translational potential of the current data, suggesting that early rhythm-stabilizing programs could serve as low-intensity, scalable preventive tools aimed at enhancing resilience and mitigating the progression toward mood disorders. While organizational and structural factors (*e.g*., adequate staffing, professional recognition, workload management) remain essential determinants of healthcare workers’ psychological well-being, individual-level rhythm stabilization strategies may offer additional, complementary benefits. We propose a flexible, structured intervention aimed at promoting rhythm regulation, mood stabilization, and stress management. The program combines psychoeducation, practical skills, and home exercises and can be tailored to individual or group needs. Key elements include the following:

- DYMERS framework and vulnerability factors, including the brief psychoeducation on DYMERS as a heuristic model linking behavioral rhythm dysregulation to mood vulnerability [37, 46, 47], as well as discussion of genetic, environmental, and lifestyle moderators (including heritability of hyperactivity-exploratory traits) and their relevance in healthcare settings [13, 46, 48-51].
- Sleep–wake hygiene and daily routine structuring [52-58] and personal stressors and specific mood triggers [36, 59, 60].
- Stress and emotion regulation and cognitive strategies, including mindfulness and relaxation techniques [33, 61-65].
- Social and interpersonal skill development to maintain stable routines and support networks [43, 63, 66-72].
- Early warning signs monitoring and mood tracking to enhance self-awareness and proactive coping [73-77].

This approach is intended as a conceptual and heuristic framework rather than a validated clinical protocol. It highlights how rhythm-oriented interventions could complement organizational strategies (*e.g*., staffing, workload management) to reduce HCWs’ psychological distress and enhance resilience. It aims to guide both empirical research and clinical translation toward rhythm-oriented prevention and early detection strategies.

## 5 LIMITATIONS

Despite the promising findings, this study has several limitations that should be acknowledged.

First, the voluntary convenience sampling and relatively small sample size may limit the generalizability of the results. The recruitment strategy may have introduced self-selection bias, as individuals experiencing higher occupational stress might have been less likely to participate. Second, the cross-sectional design precludes any conclusions regarding causality or temporal dynamics, underscoring the need for longitudinal follow-up studies to examine potential trajectories toward mood or circadian rhythm disorders. Third, because participants were recruited within a single healthcare organization, it was not possible to collect certain socio-demographic data (such as years of service, department, or shift type) without compromising anonymity. While this choice ensured compliance with privacy and ethical principles, it limits the ability to explore potential moderators or sources of variability in the outcomes. Fourth, the reliance on self-reported measures may have introduced response bias and measurement inaccuracy, particularly concerning subjective experiences of rhythm dysregulation or mental health symptoms.

Finally, this study represents a secondary and exploratory analysis within a broader research framework, primarily intended to generate hypotheses and identify potential risk factors for mental health vulnerability among healthcare workers. Within this heuristic perspective, a preliminary preventive intervention model was proposed not as a validated clinical tool, but as a conceptual framework aimed at guiding future empirical research and translational efforts toward the development of evidence-based prevention and early intervention strategies for this at-risk population.

## CONCLUSION

While this single-center study conducted within the Italian healthcare context limits external validity, the findings contribute to understanding rhythm dysregulation as a potential early indicator of mental health vulnerability in healthcare workers. Future research should include larger and more heterogeneous samples, adopt longitudinal designs to monitor progression toward mood disorders, and rigorously evaluate the effectiveness of rhythm regulation interventions within preventive psychiatry frameworks. The integration of digital tools for continuous and objective monitoring of social and biological rhythms may further enhance assessment precision and personalization.

From an institutional perspective, these findings also align with Italian occupational health policies, which mandate preventive and educational actions to safeguard workers’ psychological well-being. Incorporating rhythm-stabilizing and mental health literacy programs within workplace training could therefore represent a feasible and legally supported avenue for early prevention in healthcare settings [78].

## Figures and Tables

**Table 1:** Characteristics of the sample, frequency of depressive episodes, and BRIAN score.

-	HCWS (N=97)	PHQ<10 N=63 (64.95%)	PHQ>9 N=34 (35.05%)	STAT
**Men and Female N(%)**	29 (29.9%) 68 (70.1%)	24 (85.3%) 39 (57.35%)	5 (14.7%) 29 (42.65%)	**Depressive Episode by sex:** x^2^ = 5.764 *p*=0.016 or women = 3.57 ci95%1.2-10.5
**>49 and <50 N(%)**	36 (37.11%) 61 (62.89%)	23 (63.89%) 40 (65.57%)	13 (36.11%) 21 (34.43%)	**Depressive Episode by age:** x^2^ = 0.013 *p*=0.10 or <50 = 0.95 ci95%0.4-2.2
**Mean Age**	43.89±12.64	43.74±12.95	44.17±11.50	1,95df f=0.026, *p*=0.872
**Medical Doctors and Not Medical N(%)**	35 (36.08%) 62 (63.92%)	27 (77.14%) 36 (51.06)	8 (22.86%) 26 (48.94%)	**Depressive Episode by role:** x^2^ = 3.577 *p*=0.059 or notmd = 2.44 ci95%1.0-6.2
**Mean Brian Score**	46.14±10.96	41.29±12.58	55.12±7.94	**Difference between depressed and not depressed:** 1,95df f=37.740 *p*<0.0001
**Men and Female Mean Brian Score**	42.62±9.68 47.64±12.82	40.01±10.88 41.83±13.30	52.60±3.88 55.55±8.64	**Difference by Sex:** 1,95df f=3.569 *p*=0.062

**Table 2:** Comparison with a published study on the BRIAN Score in clinical and non-clinical samples in the same area.

N(%)	Stabilized Psycosys [31] (N=51)	BD [32] (N=40)	Community Sample [27] (N=82)	BD [33] (N=64)
**Men**	40 (65.57%)	12 (30%)	32 (39.0%)	21 (32.8%)
**Mean Age**	37.22±10.7	47.23 ± 13.37	44.63±12.95	47.23 ± 13.37
**Brian Score**	40.40±7.02	45.07 ± 12.00	22.22±11.19	48.97±12.54
**Brian Score**	43.70±10.36	46.29±9.76	44.77±11.59	45.99±10.71
**HCWS Standardized**				
**ANOVA 1 Way**	1,146 df	1,135 df	1,177 df	1,159 df
	f=4.162	f=0.386	f=173.602	f=2.603
	*p*=0.043	*p*=0.536	*p*<0.0001	*p*=0.109
	d=0.37		d=1.97	

## Data Availability

All data generated or analyzed during this study are included in this published article.

## LIST OF ABBREVIATIONS

HCWs: Healthcare Workers
DYMERS: Dysregulation of Mood, Energy, and Social Rhythms
PHQ-9: Patient Health Questionnaire-9
BRAIN: Biological Rhythms Interview of Assessment in Neuropsychiatry
IPSRT: Interpersonal and Social Rhythm Therapy
PTSD: Post-Traumatic Stress Disorder
ADHD: Attention Deficit/ Hyperactivity Disorder

## STANDARDS OF REPORTING

STROBE guidelines were followed.
